# Biologic activity of the novel small molecule STAT3 inhibitor LLL12 against canine osteosarcoma cell lines

**DOI:** 10.1186/1746-6148-8-244

**Published:** 2012-12-17

**Authors:** Jason I Couto, Misty D Bear, Jiayuh Lin, Michael Pennel, Samuel K Kulp, William C Kisseberth, Cheryl A London

**Affiliations:** 1 Department of Veterinary Biosciences, The Ohio State University, Columbus, OH, 43210, USA; 2 Department of Pediatrics, College of Medicine, The Ohio State University, Columbus, OH, 43205, USA; 3 Comprehensive Cancer Center, The Ohio State University, Columbus, OH, 43210, USA; 4 Department of Veterinary Clinical Sciences, The Ohio State University, Columbus, OH, 43210, USA; 5 College of Public Health, The Ohio State University, Columbus, OH, 43210, USA; 6 Department of Medicinal Chemistry, College of Pharmacy, The Ohio State University, Columbus, OH, 43210, USA

**Keywords:** STAT3, Osteosarcoma, Canine

## Abstract

**Background:**

STAT3 [1] has been shown to be dysregulated in nearly every major cancer, including osteosarcoma (OS). Constitutive activation of STAT3, via aberrant phosphorylation, leads to proliferation, cell survival and resistance to apoptosis. The present study sought to characterize the biologic activity of a novel allosteric STAT3 inhibitor, LLL12, in canine OS cell lines.

**Results:**

We evaluated the effects of LLL12 treatment on 4 canine OS cell lines and found that LLL12 inhibited proliferation, induced apoptosis, reduced STAT3 phosphorylation, and decreased the expression of several transcriptional targets of STAT3 in these cells. Lastly, LLL12 exhibited synergistic anti-proliferative activity with the chemotherapeutic doxorubicin in the OS lines.

**Conclusion:**

LLL12 exhibits biologic activity against canine OS cell lines through inhibition of STAT3 related cellular functions supporting its potential use as a novel therapy for OS.

## Background

The Signal Transducers and Activators of Transcription (STATs) are a family of cell signaling proteins that play critical roles in inflammation, proliferation and differentiation [1-3]. The STAT family is comprised of 7 isoforms with a variety of unique but also overlapping functions. STAT proteins play critical roles in responding to extracellular signals from growth factors and cytokines, as well as regulating gene transcription in the nucleus. STAT3 in particular has been shown to be dysregulated in many cancers including osteosarcoma (OS) and is frequently associated with malignant transformation and resistance to apoptosis in other tumor types [4-6].

In the normal cell, activation of cell surface receptors induces phosphorylation of specific tyrosine residues on STAT3, either through activation of receptor tyrosine kinase’s (RTKs) or janus kinases (JAKs), depending on the nature of the signaling stimulus. The phosphorylated STAT3 (pSTAT3) molecules then homodimerize via their SH-2 domains and subsequently translocate into the nucleus where binding to promoter elements of target genes acts to regulate their transcription [7,8]. While STAT3 activation is transient in normal cells due to a host of endogenous protein regulators (e.g., PIAS, SOCS), neoplastic cells often display constitutive STAT3 activation, which contributes to increased angiogenesis, metastasis and chemotherapy resistance [9,10].

Although originally discovered as a protein involved in the pathway transducing a signal in response to interferon [11], STAT3 was not linked to cancer until it was shown to be essential for *v-src* mediated cellular transformation [12]. The importance of STAT3 in tumor progression and survival is supported by the fact that overexpression of pSTAT3 has been linked to poor prognosis in several cancers and as such, has been proposed as a relevant target for therapeutic intervention [13-15].

Our work and that of others has demonstrated that both human and canine OS cell lines and tumors constitutively express pSTAT3 and as such, STAT3 represents a potential therapeutic target for this disease [4,13,16]. The identification of novel therapeutic targets for OS is critical given that approximately 40% of children and over 90% of dogs will die from OS [17,18]. To this end, several small molecule STAT3 inhibitors have been developed and some have shown promising activity both *in vitro* and in mouse xenograft models [19-21]. However, most of these inhibitors have suffered from issues such as poor solubility that preclude their clinical development. Using structure based design, we have developed LLL12 as a non-peptide small molecule inhibitor of STAT3 that possesses good solubility and predictable oral bioavailability [20]. LLL12 binds to the phosphorylated tyrosine on STAT3 monomers, blocking dimerization and subsequent translocation into the nucleus, abrogating its function as a transcription factor. The purpose of this study was to characterize the biologic activity of this new STAT3 inhibitor, LLL12, in canine OS cells and evaluate its ability to inhibit STAT3 and its downstream targets.

## Methods

### Cell lines and reagents

Canine OS cell lines OSA 8 and OSA 16 were provided by Jaime Modiano (University of Minnesota, Minneapolis, MN), the canine D17 OS cell line was purchased from American Type Cell Culture Collection (ATCC, Manassas, VA), and the canine Abrams OS cell line was provided by Doug Thamm (Colorado State University, Fort Collins, CO). OSA 8, OSA 16 and D17 were maintained in RPMI-1640 supplemented with 10% FBS, non-essential amino acids, sodium pyruvate, penicillin, streptomycin, L-glutamine, and HEPES (4-(2-hydroxythyl)-1-piperazineethanesulfonic acid) at 35°C, supplemented with 5% CO_2_. The Abrams cell line was cultured in DMEM medium with 10% FBS and L-glutamine. Normal canine osteoblasts (Cell Applications Inc, San Diego, CA) were cultured in canine osteoblast medium (Cell Application Inc). LLL12 was synthesized and purified as described previously [20]. The following antibodies were used for Western blotting experiments: pSTAT3 (Y705, Cell Signaling Technology, Danvers, MA), total STAT3 (Cell Signaling Technology), survivin (Novus Biologicals, Littleton, CO) and β-actin (Santa Cruz Biotechnology, Santa Cruz, CA).

### Cell proliferation

OS cells (2.5 × 10^3^) were seeded in triplicate in 96-well plates overnight in 10% FBS supplemented medium and incubated with DMSO or increasing concentrations of LLL12, doxorubicin, or both for 24 hours. The medium was removed and the plates were frozen at −80°C overnight before processing with the CyQUANT® Cell Proliferation Assay Kit (Molecular Probes, Eugene, OR) according to the manufacturer’s instructions. Cell proliferation was calculated as a percentage of the DMSO-treated control wells and IC_50_ values derived after plotting proliferation values on a logarithmic curve. Each experiment was repeated 3 times.

### Detection of apoptosis

OS cells (1.1×10^4)^ were seeded in triplicate in 96-well plates overnight in 10% FBS supplemented medium and incubated with medium only, DMSO or LLL12 at increasing concentrations for 24 hours. Caspase 3/7 activity was determined using the SensoLyte® Homogeneous AMC Caspase 3/7 Assay kit (Anaspec Inc, San Jose, CA) according to manufacturer’s instructions. To further assess apoptosis, 2×10^6^ cells were plated in a T175 plate and allowed to grow overnight before being treated with DMSO or LLL12 (0.5 μM) for 24 hours. The cells were then harvested and incubated with FITC conjugated Annexin V and propidium iodide dye (PI) following the manufacturer’s protocol (BD Biosciences, San Jose, CA) before evaluation by flow cytometry (FACS Caliber, BD Biosciences). CellQuest software (BD Biosciences) was used to analyze the samples for early and late apoptosis.

### Western blotting

OS cells or canine osteoblasts (2×10^6^) in 1% FBS medium were treated with DSMO or 0.5 μM LLL12 for 12 hours. Normal canine osteoblasts were serum starved for 2 hours prior to identical treatment. Protein lysates were prepared and quantified, separated by SDS-PAGE, and Western blotting was performed using previously described methods [4]. The membranes were incubated overnight with anti-pSTAT3 (Y705, Cell Signaling Technology, Danvers, MA) or anti-survivin (Novus Biologicals, Littleton, CO) antibodies, then incubated with appropriate horseradish peroxidase linked secondary antibodies, washed, and exposed to substrate (SuperSignal West Dura Extended Duration Substrate, Pierce, Rockford, IL). Blots were stripped, washed, and reprobed for total STAT3 (Cell Signaling Technology) or β-actin (Santa Cruz Biotechnology, Santa Cruz, CA), respectively.

### RT-PCR and qRT-PCR

Total RNA was extracted from canine OS cells in 10% FBS supplemented medium following 12 hours of treatment with DMSO or 0.5 μM LLL12 using RNeasy Mini Kits (Qiagen, Valencia, CA) according to the manufacturer’s instructions. After RNA extraction, samples were treated with DNase I using RQ1 Rnase-Free DNase (Promega, Madison, WI). cDNA was generated from 2 μg of total RNA using Superscript III reverse transcriptase kit (Invitrogen, Carlsbad, CA) according to the manufacturer’s instructions. For each PCR reaction, 1/20 of the resultant cDNA was used in a total volume of 25 μl. Primers designed and utilized for canine survivin, cyclin D1, BCL-2, VEGFA, MCL-1 and 18 s are listed in Table 1, as are the annealing temperatures for each reaction. Standard PCR was performed with all primer sets and amplicon length verified through agarose gel electrophoresis and visualization of products using the Alpha Imager system (Alpha Innotech Corp, San Leandro, CA). 

**Table 1:** Primers for canine reverse transcriptase polymerase chain reactions

**Primers**	**Primer sequences**	**Tm°**
Canine Survivin F	5^′^- GAA GGC TGG GAG CCA GAT GAT G -3^′^	66.4
Canine Survivin R	5^′^- CGC ACT TTC TTT GCG GTC TC -3^′^	62.4
Canine Cyclin D1 F	5^′^- GTC TGC GAG GAG CAG AAG T -3^′^	62.3
Canine Cyclin D1 R	5^′^- GAG GAA GTG CTC GAT GAA GT -3^′^	60.6
Canine BCL-2 F	5^′^- GAG CAG CCA CAA CCG GAG AGT C -3^′^	68.3
Canine BCL-2 R	5^′^- CGG ATC TTT ATT TCA CGA GGC AC -3^′^	62.8
Canine MCL-1 F	5^′^- CAA CCA CGA GAC AGC CTT CCA AG -3^′^	62.6
Canine MCL-1 R	5^′^- CAC TGA AAA CAT GGA CAA TCA C -3^′^	58.9
Canine 18s F	5^′^- AAA TCC TTT AAC GAG GAT CCA TT -3^′^	57.4
Canine 18s R	5^′^- AAT ATA CGC TAT TGG AGC TGG A -3^′^	58.9

To quantitatively measure the effect of LLL12 treatment on STAT3 downstream targets, total RNA was collected as described above. Real-time quantitative PCR was performed using Applied Biosystem’s StepOne Plus Real-Time PCR system (Applied Biosystems, Foster City, CA). Canine survivin, cyclin D1, BCL-2, VEGFA, MCL-1 and 18 s mRNA were detected using Fast SYBR green PCR master mix (Applied Biosystems) according to the manufacturer’s protocol. All reactions were performed in triplicate and included non-template controls for each gene. Relative expression was calculated using the comparative threshold cycle method [22]. Experiments were repeated 3 times using samples in triplicate.

### Drug combination analysis

Experiments were performed in 96-well plates. OS cells were seeded at a density of 2.5×10^4^ cells per well in RPMI medium containing 10% FCS. Stock solutions of LLL12 and doxorubicin were generated and serial dilutions (2-fold) for each compound were prepared, with the concentration range from .0625X to 4X the IC_50_ value of each drug. To assess potential synergistic interactions, the treatment regimen involved simultaneous treatment of cells with LLL12 and doxorubicin for 24 hours, in addition to controls consisting of cells treated with the individual compounds alone for 24 hours. All treatments were performed in triplicate wells. Following drug treatment, the number of viable cells in each well was determined using CyQUANT® as described previously. Drug interactions were analyzed using CompuSyn 3.0.1 (ComboSyn, Inc.,Paramus, NJ), which is based on the median effect model of Chou and Talalay [23].

### Statistical analysis

All the values reported are mean ± SD. Delta CTs from qRT-PCR were compared using two sample t-tests and Holm’s method [24] was used to control type-I error across tests of multiple genes. The Jonckhere-Terpstra (JT) test [25,26] was used to test for a monotone trend in cell proliferation and caspase activity with dose of drug. If the JT test was insignificant, we performed the Mack-Wolfe test [27] for a non-monotone, or umbrella, dose–response. All analyses were performed using SAS Version 9.2 (SAS Inc., Cary, NC). The Mack-Wolfe test was performed using the MWUSPU and MWUSPK SAS macros developed by Juneau [28].

## Results

### LLL12 Inhibits the proliferation of canine OS cell lines

**Figure 1:**
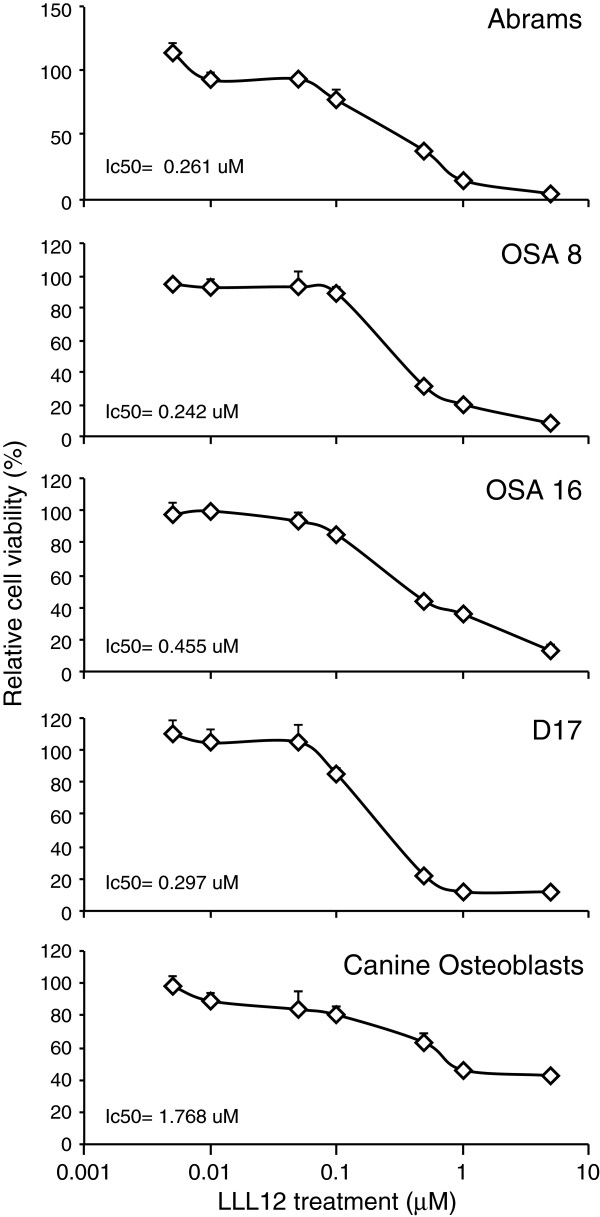
**Effects of LLL12 on the proliferation of canine OS cell lines and normal osteoblasts. **Canine OS cell lines (Abrams, OSA 8, OSA 16 and D17) and normal canine osteoblasts were treated with vehicle or LLL12 for 24 hours. Proliferation was analyzed using the CyQUANT® cell proliferation assay kit. Proliferation values are listed as a percentage of DMSO control. Experiments were performed in triplicate and repeated three times. For each cell line, there was a significant decreasing trend in cell proliferation with dose of LLL12 (p < 0.001).

Canine OS cell lines were treated with increasing concentrations of LLL12 (0.05 μM- 5 μM) for 24 hours and effects on cell proliferation were assessed. LLL12 significantly reduced cell proliferation at concentrations as low as 0.1 μM with the calculated IC_50_ concentrations in the nanomolar range (231–411 nM) for all cell lines (Figure 1). Normal canine osteoblasts were comparatively resistant to the anti-proliferative effects of LLL12, with an approximately 7-fold higher calculated IC_50_ of 1.780 μM (Figure 1).

### LLL12 Promotes apoptosis of canine OS lines

**Figure 2:**
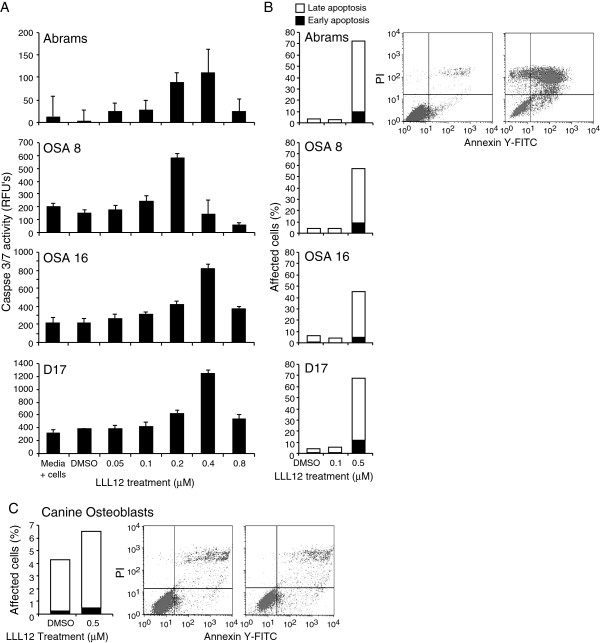
**Evaluation of canine OS cell lines for apoptosis following LLL12 treatment. **Canine OS cell lines treated with vehicle or LLL12 for 24 h. Apoptosis was assessed by measuring activated caspases 3 and 7 (**A**) using the Sensolyte® Homogeneous AMC Caspase-3/7 Assay kit. Experiments were performed in quadruplicate and repeated three times. The same canine OS cell lines were treated under identical conditions as above and stained with annexin V-FITC/PI and analyzed by flow cytometry (**B**). Normal canine osteoblasts were treated under identical conditions and stained as above (**C**). There was a significant increasing trend in caspase activity for all lines except OSA 8 (p<0.01).

To determine if LLL12 growth inhibition was mediated via apoptosis, canine OS cell lines were treated with DMSO or LLL12 for 24 hours, and caspase 3/7 activity was measured. In all cell lines, caspase 3/7 activity was increased at 24 hours post treatment with LLL12 at concentrations of 0.4-0.8 μM (Figure 2A). OS cells were also stained with Annexin V-FITC/PI and analyzed by flow cytometry to assess the percentage of early and late apoptotic cells in the population. After a 24 hour exposure to 0.5 μM LLL12 there was an increase in the proportion of early apoptotic (Annexin V positive, up to 22-fold increase) and late apoptotic (Annexin V/PI positive, up to 13-fold increase) cells. This correlated with data generated from the caspase assay (Figure 2B). Normal canine osteoblasts were treated and analyzed by flow cytometry as described above, and were far less sensitive to the apoptosis inducing effects of LLL12 (Figure 2C).

### LLL12 Treatment decreases pSTAT3 and survivin expression in canine OS lines

**Figure 3:**
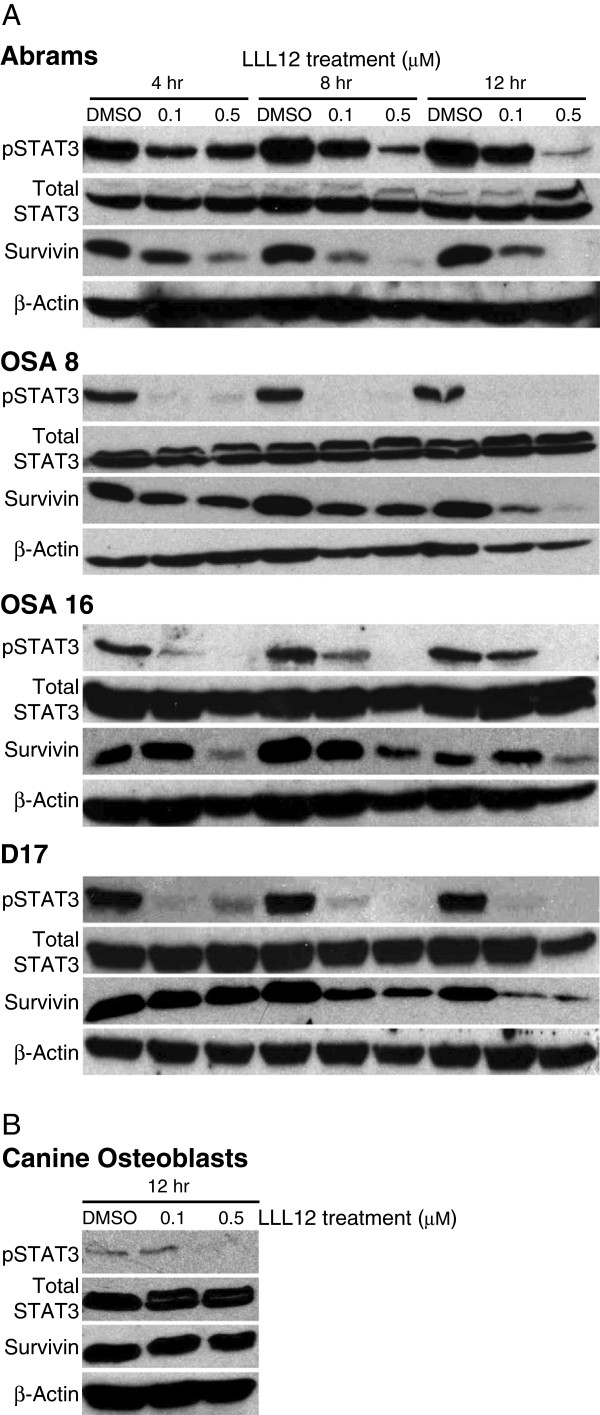
**Analysis pSTAT3, STAT3 and survivin in canine OS cell lines following LLL12 treatment. **Canine OS cell lines were treated with DMSO or LLL12 for 4, 8 and 12 hours prior to collection. Normal canine osteoblasts were treated with DMSO or LLL12 for 12 hours prior to collection. Protein lysates were generated and separated by SDS-PAGE and Western blotting for pSTAT3, STAT3, survivin and β-actin were performed. Experiments were repeated two times.

Canine OS cells and normal canine osteoblasts were treated with DMSO, 0.1 μM LLL12 or 0.5 μM LLL12 for 4, 8 or 12 hours to determine the time and dose dependence of its effect on STAT3 phosphorylation and survivin expression. Western blot analysis revealed pSTAT3 was completely downregulated following treatment with 0.5 μM LLL12 for only 4 hours, with a concomitant downregulation of survivin expression (Figure 3A). As expected, these results were time- and dose-dependent. Importantly, normal canine osteoblasts treated identically to their OS counterparts had significantly lower pSTAT3 expression and demonstrated no change in survivin expression (Figure 3B) following 0.5 μM LLL12 treatment at 12 hours.

### LLL12 Treatment decreases STAT3-mediated gene transcription

**Figure 4:**
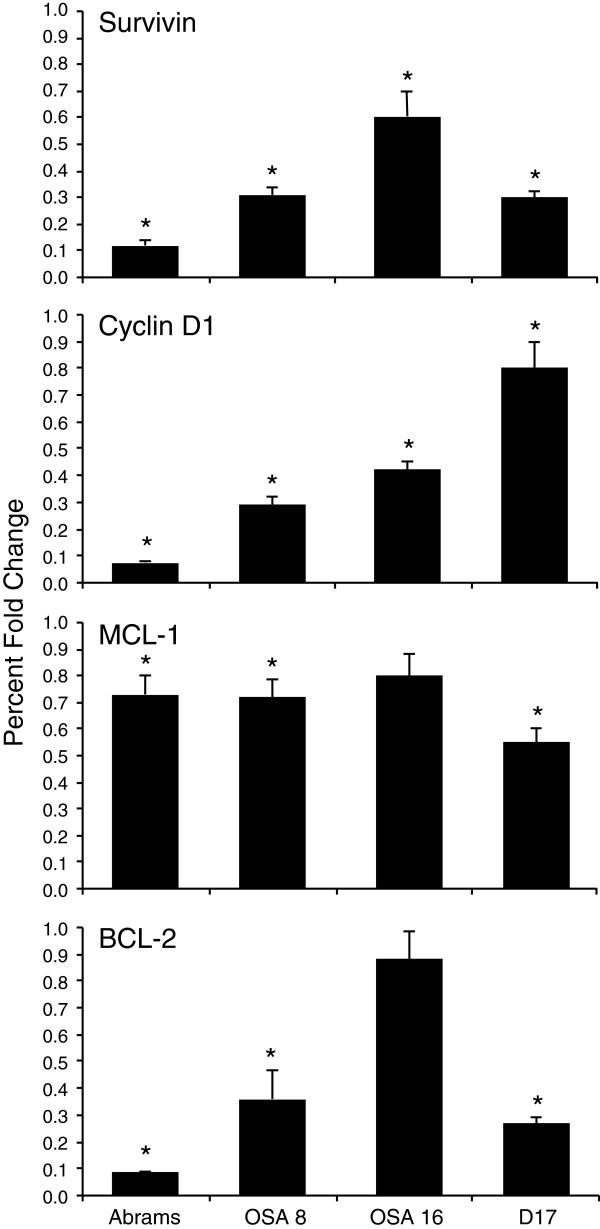
**Evaluation of STAT3-related gene expression using qRT-PCR after LLL12 treatment. **Canine OS cell lines were treated with DMSO or 0.5 μM LLL12 for 12 hours. RNA was collected and qRT-PCR was performed for survivin, cyclin D1, BCL-2 and MCL-1 and 18 s. Experiments were performed in triplicate and repeated three times. Delta CTs from qRT-PCR were compared using two sample t-tests and Holm’s method was used to control type-I error across tests of multiple genes (**p<0.02, *p<0.001).

To assess the effects of LLL12 on transcriptional targets of STAT3 the expression of cyclin D1, BCL-2, MCL-1 and survivin was assessed using quantitative RT-PCR. Standard PCR was run with all primer sets and amplicon length verified prior to quantitative analysis. Expression of the STAT3 regulated genes evaluated was significantly downregulated in all 4 OS cell lines after 12 hours of treatment with 0.5 μM LLL12 when compared to DMSO treated cells (Figure 4) supporting the notion that inhibition of pSTAT3 by LLL12 affects its transcriptional activity.

### LLL12 Enhances the antiproliferative effects of doxorubicin in canine OS cells

**Figure 5:**
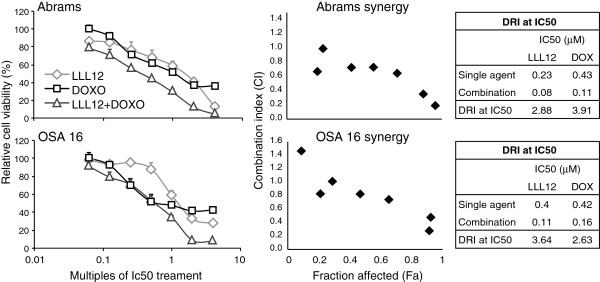
**LLL12 synergizes with doxorubicin in canine OS cell lines. **Proliferation curves and combination index graphs of canine (Abrams and OSA 16) OS cell lines after 24 hours of treatment with LLL12, doxorubicin, or both. For each cell line and treatment (LLL12 alone, DOXO alone, LLL12 + DOXO) there was a significant decreasing trend in cell proliferation with dose (*p < 0.001).

To assess whether inhibition of pSTAT3 would enhance the biologic activity of chemotherapy in OS cell lines, Abrams and OSA 16 cells were treated with LLL12 (0.016 μM-1 μM), doxorubicin (0.022 μM-1.4 μM) or both drugs in combination over a range of doses reflecting multiple concentrations of their respective IC_50_ concentrations ranging from 0.0625× to 4×. Dose–response curves and Combination Index (CI) graphs were generated and analyzed using Compusyn software (Figure 5). The CI values were <1 in 12/14 dose combinations in both OS cell lines tested demonstrating that LLL12 exhibits synergistic anti-proliferative effects with doxorubicin in these lines. The dose reduction index (DRI), which determines the magnitude of dose reduction allowed for each drug when given in synergistic combination, as compared with the concentration of a single agent that is needed to achieve the same effect was 2.63-3.91 for LLL12 and doxorubicin in the OS lines. These data further support the notion that LLL12 and doxorubicin interact in a synergistic manner in OS cell lines.

## Discussion

Despite some advances in our understanding of the underlying molecular biology of OS, treatment for this disease has not changed significantly over the last 15 years in dogs or people [29]. Surgical resection and aggressive chemotherapy protocols are effective, but have failed to improve the 5-year overall survival rate past 60-70% in humans [18]. Similarly, in dogs, limb amputation followed by adjuvant chemotherapy with doxorubicin or carboplatin results in a 1-year survival rate of less than 50% and a 2-year survival rate of approximately 10-20% [30]. Treatment with doxorubicin and platinum based compounds, the current standards of care in the field, also come with the potential for significant toxicities including myelosuppression, gastrointestinal toxicity, cardiotoxicity and in humans including ototoxicity and secondary malignancies [31]. This is particularly relevant for pediatric patients in whom late toxicities can substantially affect quality of life. Clearly, new drugs and new therapeutic targets are needed to both improve the outcome of patients suffering from OS and to reduce the long-term toxicities associated with the current standard of treatment.

Our laboratory previously characterized constitutive STAT3 activation in primary canine OS tumor samples and canine OS cell lines, and showed that direct downregulation of STAT3 protein expression in OS lines using siRNA induced loss of cell viability and apoptosis [4,19]. We similarly demonstrated that two earlier STAT3 small molecule inhibitors (LLL3 and FLLL32, both developed at OSU) also impacted OS cell viability and induced cell death in all cell lines evaluated [4,19]. In concordance with our work, STAT3 dysregulation has been demonstrated in OS in humans, where high levels of STAT3 correlated with metastasis and lower rates of overall survival [13,32]. Together, these data define STAT3 as an important target for therapeutic intervention in OS, particularly given the fact that STAT3 function is dispensable in many normal cells.

The mechanism(s) of persistent STAT3 phosphorylation remain to be elucidated in OS. Constitutive STAT3 activation does not appear to occur through direct mutation in STAT3 as it does with other known oncogenes [10]. However, there are a multitude of ligands (e.g. IL-6, OSM, EGF, HGF, IGF) and kinases (RTKs, JAKs, SRC family members) that initiate STAT3 activation and thus there are many potential upstream drivers that could contribute to the observed dysregulation [9]. In our prior studies we identified OSM as a potential driver of STAT3 phosphorylation in canine OS tumor cells and found that inhibition of STAT3 signaling disrupted OSM induced biologic activities [33]. It is also possible that a loss of STAT3 regulatory mechanisms may play a role in sustained STAT3 pathway signaling.

STAT3 may also play a critical role in chemoresistance in a number of cancer types, including OS [34,35]. The mechanisms through which this may occur are not well understood, although available data suggests that upregulation of the drug-resistance and anti-apoptotic STAT3-regulated genes survivin, MCL-1 and MDR1 may play a part. Indeed, research has shown that P-gp, the product of the MDR1 gene, can have its expression mediated by STAT3 [36], providing a possible mechanism for STAT3-mediated chemotherapy resistance.

Experimental evidence generated by our laboratory and by others has clearly demonstrated that disruption of STAT3 signaling inhibits the survival and proliferation of OS cell lines and decreases the growth of OS in mouse models of disease [19,37,38]. However, the challenge has been to develop a STAT3 inhibitor that has good potential for future clinical application. LLL12 is an optimized analog of LLL3, a novel small molecule allosteric STAT3 inhibitor that has been shown to inhibit proliferation and induce apoptosis in various cancer cell lines *in vitro* and in several mouse xenograft models, including OS [20,39,40]. LLL12 works by binding to STAT3 monomers at the phosphorylation site on Y705 and thereby preventing STAT3 dimerization and translocation into the nucleus. Similarly, anti-STAT3 therapies, such as dominant negative STAT3 molecules, RNA interference and antisense oligonucleotides have been shown to be effective against a number of tumor types *in vitro*, but have yet to be tested in clinical trials, due in part to drug delivery issues including cell permeability, stability and solubility of the DNA, RNA and small molecules. With respect to the small molecule inhibitors previously tested in canine OS, FLLL32 suffered from lower activity than LLL12 and solubility issues that precluded its further use in clinical trials and LLL3, while potent, did not bind directly to the pY705 binding site of the STAT3 monomer, unlike its optimized analog LLL12, which has a 10-fold increase in simulated binding energies to STAT3 [40].

Our collaborator (J.Lin) has demonstrated that LLL12 has no off-target effects at concentrations used in this work (less than 1 μM, data not shown), and does not inhibit any of the other STAT family members [41]. Our current work shows that LLL12 inhibits cell viability while inducing apoptosis in canine OS cell lines expressing elevated levels of pSTAT3. LLL12 is quite potent, with IC50^′^s for the 4 canine OS lines between 0.23 μM and 0.41 μM. Importantly, the IC50 generated for normal canine osteoblasts was 1.78 μM, which demonstrates minimal toxicity in cells that lack constitutive activation of STAT3. With respect to the concentrations of drug used in these studies, preliminary pharmacokinetic data generated in mice indicate that exposures above 1 μM occur following intravenous and intraperitoenal administration of LLL12 (J. Lin, data not shown). Doxorubicin administration to dogs results in peak plasma levels of drug ranging from 1.3-1.5 μM, with drug concentrations above 0.2-0.4 μM lasting for 10–12 hours following a single IV bolus of drug given over 20 minutes [42]. For the agents in combination, the IC50 of LLL12 is reduced from 0.23-0.4 μM to 0.08-0.11 μM which are concentrations that are achievable in vivo; the IC50 of doxorubicin is reduced from 0.42-0.43 μM to 0.11-0.16 μM, which are also concentrations achievable in vivo. Therefore, we believe the drug concentrations used in this body of work are reflective of exposures obtainable in vivo.

STAT3 transcriptional targets were all downregulated after only 12 hours of 0.5 μM LLL12 treatment, showing clear, rapid effects at biologically relevant concentrations. Protein expression of pSTAT3 and survivin were similarly downregulated under identical conditions. While the timing of survivin downergulation lagged somewhat behind the loss of pSTAT3, this was expected as STAT3 is a transcriptional activator of survivin and existing transcript and protein would need to turn over first before a loss of survivin protein would be observed. Additionally, normal canine osteoblasts which have little to no pSTAT3 exhibit no loss of survivin protein at 12 hours of treatment, supporting the notion that STAT3 and not other transcription factors are linked to the loss of survivin. Together, these results show that LLL12 is more potent at inhibiting cell proliferation and decreasing pSTAT3 protein expression than both LLL3 and FLLL32.

The synergy experiments in combination with doxorubicin show promise, with obvious clinical implications. LLL12 has strong activity against cells with constitutive pSTAT3 expression, but little effect on normal cells. The significant dose reduction index seen when LLL12 is used with doxorubicin could permit its use in the setting of lower doxorubicin doses, thereby potentially limiting some of the acute and long-term toxicities associated with dose intense doxorubicin. This has particular relevance for dogs where cardiotoxicity limits the cumulative dose of doxorubicin to 180 mg/m2 (typically 6 doses) and the pediatric population where cognitive deficiencies, secondary neoplasia, and/or cardiac disease occur as long-term consequences following dose-intense treatment with doxorubicin.

## Conclusions

LLL12, a novel allosteric STAT3 inhibitor, inhibited proliferation and promoted apoptosis in canine OS cell lines. LLL12 decreased pSTAT3 and survivin expression and downregulated the STAT3-mediated gene transcription of survivin, cyclin D1, BCL-2 and MCL-1 within 12 hours of drug exposure in the nanomolar range. These data support the clinical development of LLL12 for the treatment of OS and other cancers in which STAT3 is known to be constitutively activated.

### Ethical Support

All the studies we performed were in vitro with cell lines and as such, no IACUC or approval was necessary. The cell lines have been available for several years and were previously published.

## Competing interests

The authors declare that they have no competing interests.

## Authors’ contributions

JC carried out molecular experiments on OS cell lines and drafted the manuscript. MB participated in RT-PCR design and performance, as well as optimizing the experimental design for all the molecular experiments. JL provided LLL12. WK assisted in experimental design. SK performed the analysis of syngergy experiments. MP designed and performed all statistical tests. CL conceived the study, assisted in experimental design, and helped draft the manuscript. All authors read and approved the final manuscript.
